# Efficacy of docusate in the treatment of constipation in pediatric patients

**DOI:** 10.3389/fped.2025.1652620

**Published:** 2025-10-10

**Authors:** Hamsah Saif, Tanay Maddula, Kerry Mendelsohn, Meredith Akerman, Nicole Sweeney, Estela Noyola, Gladys El-Chaar

**Affiliations:** ^1^ NYU Langone Hospital Long Island, Mineola, NY, United States; ^2^ Department of Pharmacy Practice, St John’s University College of Pharmacy and Health Sciences, Queens, NY, United States; ^3^ NYU Grossman Long Island School of Medicine, Mineola, NY, United States

**Keywords:** docusate, PEG-3350, constipation, pediatric hospital, laxative

## Abstract

**Objectives:**

We hypothesized that docusate is effective in the treatment of constipation in pediatric patients. Secondary outcomes included the safety and acceptance of docusate as well as the efficacy, safety and acceptance of PEG-3350 in the treatment of constipation.

**Methods:**

This multicenter retrospective study included children 1 month to 18 years of age who received either oral docusate or PEG-3350 during their hospital admission. We documented the occurrence of bowel movements within the first 72 h of drug administration. We also evaluated time to first bowel movement, frequency of bowel movements per 24-hour periods, adverse effects and acceptance of docusate/PEG-3350 by the patients, concomitant medications, and response according to medical history.

**Results:**

There were 90 patients in each of the docusate and PEG-3350 groups. Bowel movements occurred within 72 h in 66.67% in the docusate group and 71.11% in the PEG-3350 group. There was no significant difference between the groups (*p* = 0.5196). The time to achieve first bowel movement was not different between groups (48.9 h vs. 45.4 h, docusate and PEG-3350, *p* = 0.3283). There were no differences in adverse effects or acceptance between groups.

**Conclusions:**

This is the first study that proves the efficacy of oral docusate in the treatment of hospitalized pediatric patients with acute constipation. It is also the first study that shows no difference in efficacy between docusate and PEG-3350 in pediatric patients. We hope a prospective trial would further confirm our findings.

## Highlights

• This is the first study examining the efficacy of docusate and the first study comparing docusate and PEG-3350 in pediatric patients with constipation.

• The efficacy of docusate in the treatment of constipation has been called into question in adults. There are no studies of oral docusate in children. Similarly, there are no data comparing PEG-3350 and docusate in pediatric patients.

• This is the first study that shows the efficacy and safety of docusate for the treatment of constipation in pediatric patients. This is also the first study that shows comparable efficacy of docusate and PEG 3350 in pediatric patients.

## Introduction

Constipation is a common condition in pediatric patients, with an estimated prevalence of 0.7% to 29.6% worldwide (1). It is characterized by hard and bulky stools, infrequent and painful defecation, and/or abdominal pain (2). This condition can cause significant distress to patients, negatively impact their quality of life, and significantly burden healthcare resources (3). There are many causes of constipation including, but not limited to gastrointestinal diseases, neurological disorders, other systemic diseases, surgeries and procedures, and certain medications (2, 4). Irrespective of the etiology of constipation, pharmacological interventions may be required for symptomatic relief.

The most common pharmacological agents used in the treatment of constipation include surfactant, osmotic, and stimulant laxatives. Docusate was patented for use in 1937 prior to the FDA's mandate for drug approval process. It is classified as a surfactant stool softener and was traditionally chosen as the first-line therapy for both acute and chronic use. It is a detergent molecule, which works by reducing the surface tension of the water-oil surface of the stool, enhancing water and fat absorption; this softens the stool, thus easing the straining required in a bowel movement. Docusate also helps eliminate the pain of defecation associated with constipation and is a good choice for patients who should avoid straining (5). It is considered generally safe and tolerable and is available without a prescription in oral capsule, liquid formulations and a rectal liquid formulation. Dosing may be based on weight or as a set dose in mg (Table 1) (6).

**Table 1:** Recommended dosing of docusate and PEG-3350 based on pediatric and neonatal lexi-drugs.

Agent	Dose
Docusate (6)	Manufacturer's labeling: • Children 2 years to <12 years: 50–150 mg/day in single or divided doses • Children ≥12 years and Adolescents: 50–360 mg/day in single or divided doses
	Alternate dosing: • Weight-directed dosing: Infants and Children: o 5 mg/kg/day in 1–4 divided doses
	• Age-directed (fixed) dosing: o Infants ≥6 months and Children <2 years: 12.5 mg 3 times daily o Children ≥2 years and Adolescents: 40–150 mg/day in 1–4 divided doses; in children ≥12 years and adolescents, doses up to 500 mg/day divided may be used.
Polyethylene glycol 3350 (7)	Infants, Children, and Adolescents: Oral: 0.2–0.8 g/kg/day; up to 1g/kg have been suggested; maximum daily dose: 17 g/day.

Polyethylene glycol 3350 (PEG-3350) was approved by the FDA in 1999 is classified as an osmotic laxative. Similarly to docusate, it may be used for both acute and chronic constipation. Osmotic laxatives are poorly absorbed ions or molecules that create an osmotic gradient within the intestinal lumen. This draws water into the lumen, causing water retention in the stool and making stools soft and loose (5). PEG-3350 is available as a powder that is dissolved in 4–8 ounces of a liquid. Dosing for constipation is weight-based with a maximum of 17 grams per dose (Table 1) (7). It has been shown to be safe and effective in the treatment of constipation in children (8).

In adult patients, docusate's efficacy has been called into question (9, 10). This evidence has not been duplicated in pediatric patients (11). The literature on the efficacy of docusate in this population is limited to its use as an enema and/or in combination with other laxatives (12). As such, there has been no published literature on the use of oral docusate in the treatment of constipation in children.

The primary purpose of this study was to assess the efficacy and safety of oral docusate in the treatment of constipation in pediatric patients admitted to our health system. Secondary outcomes included the assessment of the efficacy and safety of PEG-3350 in the treatment of constipation and acceptance of both agents.

## Patients and methods

### Study design

This was a multicenter retrospective study of pediatric patients admitted to our health system between January 2009 and February 2020.

Approval of this study was obtained from our institutional review board. Study personnel may have been involved in the clinical care of the patients; however, they did not have prior knowledge about the study.

The study included pediatric patients between the ages of 1 month to 18 years who received either oral docusate or oral PEG-3350 during their hospital admission. As such this was a parallel study design. Patients were excluded if they were neonates; received docusate, PEG-3350, or any other laxative prior to admission; had a length of stay of less than 72 h; a history of constipation prior to admission, neurological impairment that might affect gastrointestinal function and defecation, Hirschsprung's disease, cystic fibrosis, neuromuscular disease, tracheostomy and/or ventilator dependence; or concomitant intake of other laxatives or stimulant medications during their hospital course. As such, the indication for laxative use was expected to be acute constipation, not fecal disimpaction. Eligible patients were classified into two groups: patients who received oral docusate and patients who received oral PEG-3350.

### Data collection

Due to a change in electronic systems at this institution, various electronic medical systems were used. Data were obtained through the Siemens (January 1, 2009–July 2016), Soarian (July 2016–October 2019) and EPIC (October 2019–February 2020) systems. We recorded patient demographic information, clinical symptoms, radiographic imaging, management, and clinical outcomes. Demographic information included patient's age, gender, ethnicity, weight (kg), current diagnosis (es), reason for constipation, and current inpatient medications. Clinical outcome measures included docusate/PEG-3350 dosage, dosage forms, times of administration, indication, time from administration of first dose to first bowel movement, frequency of bowel movements per 24-hour periods, quality of the stool if documented, adverse effects of docusate/PEG-3350, changes in bowel sounds/bowel exam, abdominal radiographs if documented, and patient's acceptance of the medication.

### Outcome variables

The primary objective was documentation of a bowel movement within 72 h of receiving an appropriate dose of docusate sodium. We chose 72 h because of the need to achieve efficacy in our acute care setting. Secondary outcomes included documentation of a bowel movement within 72 h of receiving an appropriate dose of PEG-3350 in pediatric patients; the time to achieve the first bowel movement; and safety and tolerability of docusate and PEG-3350. Subgroup analyses were performed to compare efficacy and/or adverse effects between groups.

Dosages of docusate and PEG-3350 were deemed appropriate if they were prescribed according to either the Pediatric and Neonatal Lexi-Drugs dosing (6, 7), and/or manufacturer's labeling (Table 1).

#### Statistical methods

Descriptive statistics (frequencies and percentages for categorical variables) were calculated separately by group (docusate vs. PEG-3350). The two groups were compared using the chi-square or Fisher's exact test, as deemed appropriate.

The analysis of time to first bowel movement was accomplished by applying standard methods of survival analysis, i.e., computing the Kaplan–Meier (13) product limit curves, where the data were stratified by group. In cases where the endpoint event, first bowel movement, had not yet occurred, the number of hours until last follow-up was used and considered “censored”. The two groups were compared using the log-rank test. The median rates for each group were obtained from the Kaplan–Meier/Product-Limit Estimates and their corresponding 95% confidence intervals were computed, using Greenwood's formula (14) to calculate the standard error.

The above described analyses were also performed within the docusate and PEG-3350 groups separately, using the following parameters: location, gender, age, cardiac-related past medical history, concomitant medications that might affect results such as opioids, iron, or ondansetron. A result was considered statistically significant at the *p* < 0.05 level of significance. All analyses were performed using SAS version 9.4 (SAS Institute Inc., Cary, NC).

For analysis of the primary outcome, the proposed sample size was 90 subjects in the docusate group. Based on the literature, the incidence of bowel movement/efficacy of PEG-3350 is between 56% and 62%. We proposed 50% efficacy of docusate and PEG-3350 for this study (15, 16). A sample size of 90 subjects achieves 80% power to detect a difference (P1-P0) of 0.15 using a two-sided binomial test, at a significance level of 0.05. These results assume that the population proportion under the null hypothesis is 0.5. Power analysis was conducted using PASS (NCSS, LLC, Kaysville, UT, USA).

## Results

**Figure 1:**
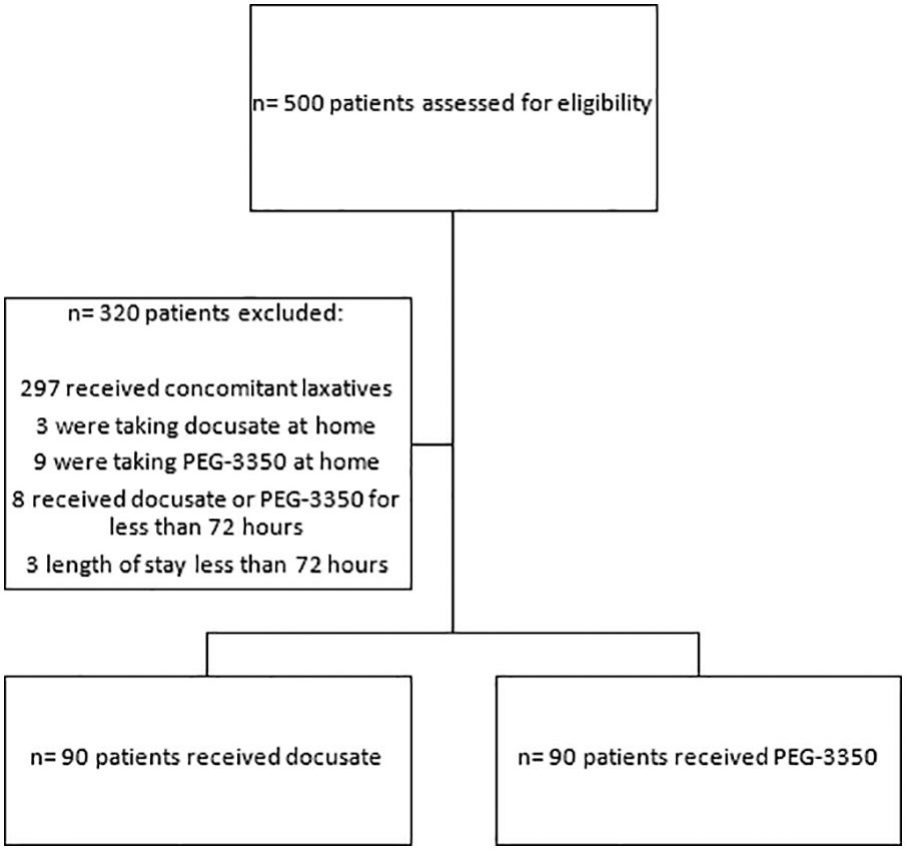
Flow chart for patient inclusion and exclusion.

Between January 2009 and February 2020, 500 patients were assessed for eligibility. Excluded from the study were 312 patients, therefore 180 patients were included (Figure 1). There were 90 patients per group. Patient demographics are described in Table 2. There were variations in the selection of the two medications according to location. There were more female patients in the PEG-3350 group compared to the docusate group (*p* = 0.295). Patients in the docusate group were older (*p* = <0.0001) and weighed more (*p* = <0.0002). There were more black patients in the docusate vs. PEG-3350 group (*p* = 0.0181). PEG-3350 was prescribed more commonly for the general indication of constipation (*p* = <0.0001), while docusate was prescribed more frequently in postoperative patients (*p* = <0.0001). The docusate group had more patients with a history of cardiovascular disease (*p* = 0.0067). The most commonly administered co-medications that could cause constipation were opioids, iron, and ondansetron. These were evenly distributed between groups. The length of stay was longer for patients who received PEG-3350 (*p* = 0.0047).

**Table 2:** Patient demographics.

Outcome		Docusate (*n* = 90)	PEG-3350 (*n* = 90)	*p*-value
Patient location, *n* (%)	Brooklyn	15 (16.67%)	0 (0%)	<0.0001
	Long Island/Winthrop	4 (4.44%)	2 (2.22%)	
	Langone Orthopedic Hospital	22 (24.44%)	9 (10%)	
	Tisch	49 (54.44%)	79 (87.78%)	
Sex, *n* (%)	Female	45 (50%)	52 (57.78%)	0.2953
	Male	45 (50%)	38 (42.22%)	
Age, years, mean ± SD		11.95 ± 6.66 [median = 14.45, IQR = (6.2, 17.5)]	7.85 ± 5.21 [median = 7.1, IQR = (3.2, 12.3)]	<0.0001
Weight, kg, mean ± SD		46.38 ± 27.36 [median = 50.05, IQR = (18, 68)]	30.3 ± 21.12 [median = 22.3, IQR = (13.4, 43.6)]	0.0002
Height, cm, mean ± SD		139.08 ± 38.28 [median = 156.2, IQR = (116.8, 162.6)]	120.85 ± 32.45 [median = 116, IQR = (92, 152.4)]	0.0003
Race, *n* (%)	Black	21 (23.33%)	8 (8.89%)	0.0181
	Native Hawaiian or other Pacific Islander	2 (2.22%)	0 (0%)	
	Other/Unknown	38 (42.22%)	44 (48.89%)	
	White	29 (32.22%)	38 (42.22%)	
Ethnicity, *n* (%)	Non-Hispanic	83 (92.22%)	79 (87.78%)	0.3203
	Hispanic	7 (7.78%)	11 (12.22%)	
Indication for laxative	Constipation	47 (52.22%)	77 (85.56%)	<0.0001
	Not documented/unknown	2 (2.22%)	0 (0%)	
	Post-op constipation	36 (40%)	13 (14.4%)	
	Post-partum constipation	5 (5.56%)	0 (0%)	
Length of stay, days, mean ± SD		13.16 ± 18.51 [median = 7, IQR = (5, 14)]	15.91 ± 20.45 [median = 9, IQR = (7, 17)]	0.0047
Concomitant medications that can cause constipation, *n* (%)		70 (77.78%)	67 (74.44%)	0.6000
Opioids, *n* (%)		57 (63.33%)	52 (57.78%)	0.4457
Iron, *n* (%)		21 (23.33%)	12 (13.33%)	0.0830
Ondansetron, *n* (%)		27 (30%)	27 (30%)	1.0000
Patients with cardio-related past medical history, *n* (%)		20 (22.22%)	7 (7.78%)	0.0067

In the docusate group, the majority of patients were dosed according to age (Table 1). Fourteen (15.6%) patients in this group did not receive recommended doses, 58 patients received capsules, 30 patients received liquid, and 2 patients received both at different times. In the PEG-3350 group, all patients received weight-based dosing, the only recommended dosing method. Ten (11.1%) in this group did not receive recommended doses, with 3 patients receiving lower and 7 patients receiving higher than recommended doses. In the PEG-3350 group, all dosage forms consisted of the powder.

### Primary and secondary clinical outcomes

The primary outcome of a bowel movement occurring within 72 h was achieved in 60 children (66.67%) in the docusate group. In the PEG-3350 group, 64 children (71.11%) achieved a bowel movement within 72 h. There was no difference between groups (*p* = 0.5196).

There were also no differences between groups in achieving a bowel movement within 24-, 48-, 96- and 120-hour periods. The time to achieve first bowel movement was not different between groups (48.9 h vs. 45.4 h, docusate and PEG-3350, *p* = 0.3283) (Table 3).

**Table 3:** Comparison of bowel movements between docusate and PEG-3350.

Variable	Docusate (*n* = 90)	PEG-3350 (*n* = 90)	*p*-value
Bowel movement within 24 h	35 (38.89%)	35 (38.89%)	1.0000
Bowel movement within 48 h	44 (48.89%)	52 (57.78%)	0.2320
Bowel movement within 72 h	60 (66.67%)	64 (71.11%)	0.5196
Bowel movement within 96 h	62 (68.89%)	67 (74.44%)	0.4082
Bowel movement within 120 h	62 (68.89%)	68 (75.56%)	0.3181
Time to first bowel movement, median (95% CI)	48.9 (25.9, 70.6)	45.4 (36.1, 50.2)	0.3283
Doses refused by patient/family	38 (42.22%)	35 (38.89%)	0.6488
Documented adverse events/ tolerability issues			
Diarrhea	6 (6.67%)	14 (15.56%)	0.0578
Loose stools	3 (3.33%)	3 (3.33%)	1.0000
Diarrhea/Loose stools	9 (10%)	17 (18.89%)	0.0898

### Subgroup analysis

There were no differences in achieving a bowel movement within 72 h according to indication for constipation, concomitant medication that could cause constipation, or past medical history of cardiovascular disease (Table 4).

**Table 4:** Bowel movement within 72 h according to indication and concomitant medications.

Indication for laxative use	Docusate (*n* = 90)	PEG-3350 (*n* = 90)	*p*-value
General constipation	**47**	**77**	
	37 (78.72%)	53 (68.83%)	0.231
Post-op constipation	**36**	**13**	
	22 (61.11%)	11 (84.62%)	0.174
Post-partum constipation	**5**	**0**	
	0 (0%)		N/A
Patients with cardio-related past medical history	**20**	**7**	
	19 (95%)	7 (100%)	1.000
Concomitant medications that can cause constipation	**70**	**67**	
	47 (67.14%)	51 (76.12%)	0.245
Opioids	**57**	**52**	
	37 (64.91%)	41 (78.85%)	0.107
Iron	**21**	**12**	
	11 (52.38%)	9 (75%)	0.278
Ondansetron	**27**	**27**	
	17 (62.96%)	19 (70.37%)	0.564

Numbers in bold indicate the number of subjects who received the laxative according to indication or who were on a concomitant medication that can cause constipation.

### Adverse events

Loose stools were reported similarly between groups (3.33% per group; *p* = 1). There were more frank diarrheal episodes in patients in the PEG-3350 group vs. the docusate group (15.56% and 6.67%, respectively; *p* = 0.0578) (Table 3).

### Acceptance

Refusal of the dose was reported in 38 (42.22%) patients in the docusate group and 35 (38.89%) patients in the PEG-3350 group (*p* = 0.6488) (Table 3).

## Discussion

This is the first study that assessed the efficacy and safety of oral docusate and the first to compare oral docusate with PEG-3350 in pediatric patients. Our primary outcome showed that oral docusate was effective in treating constipation within 72 h of starting therapy in hospitalized pediatric patients. Our secondary outcome demonstrated similar efficacy between docusate and PEG-3350 in treating constipation in our patients.

Data on the use of docusate in pediatric patients is limited to rectal administration in two trials. In 2009, Bekkali, et al. (17) conducted a study involving 90 children with mean age of 7.5 ± 2.8 years. They compared the use of PEG-3350 (1.5 g/kg/day) vs. rectally administered docusate for 6 consecutive days. After this period, successful rectal disimpaction, defecation, fecal incontinence frequencies, and behavior scores were assessed. PEG-3350, 1.5 g/kg was used, a dose indicated for disimpaction, not for the treatment of constipation (0.2–0.8 g/kg). Results showed no statistically significant difference between PEG-3350 and docusate. In this study the authors compared rectally administered docusate to a higher dose of PEG-3350. Despite the variability in rectal absorption of medications, docusate was equally effective to PEG-3350.

In 2016 Omarsson, et al. (18) compared the efficacy of high dose free fatty acid (FFA) suppositories vs. Klyx (docusate and sorbitol 70%) enema in 77 patients with ages ranging from 1 to 17 years. Time to bowel movement was shorter in the Klyx group as compared to the high dose FFA group. (*p* = 0.0003).

Given the more recent introduction of PEG-3350 in the treatment of constipation, there are more studies that have examined its efficacy and safety in pediatric patients. In prospective and retrospective trials PEG-3350 has been shown to be effective in treating constipation in 93% and 97% of children (8).

In our study patient in the docusate group were older and weighed more than children in the PEG-3350 group. More black patients received docusate. Given the retrospective nature of the study, we were unaware of the treatment decisions influencing prescribing patterns. More patients were prescribed docusate postoperatively which may be due to the need to reduce oral fluid intake in these patients. Patients who had a cardiac-related medical history were prescribed docusate more frequently, likely because of less volume requirement as well. Therefore, a potential advantage of docusate over PEG-3350 is its smaller fluid quantity required per dose, 1–10 ml of docusate compared to 240 ml (8 fl oz) of PEG-3350. This advantage can be beneficial in patients who require fluid restriction, such as patients with cardiac or renal diseases (19, 20).

A common indication for laxatives in hospitalized patients includes functional constipation, post-operative constipation, and/or medication-induced constipation. In our study, response to docusate and PEG-3350 was similar according to indication, in patients with a cardiac-related medical history or concomitant medications that could cause constipation.

While the literature describes a faster onset of action for docusate as compared to PEG-3350 (12–72 h and 24–96 h, respectively) we did not find a difference in our study (5, 7).

In the literature, diarrhea has been reported in 10%–15% of pediatric patients who received PEG-3350 (21, 22) whereas the incidence of diarrhea with docusate has not been reported. In our study, docusate and PEG-3350 were associated with diarrhea or loose stools in 10% and 18.9% of the patients, respectively. There was more diarrhea in the PEG-3350 group, however our study may not have been powered to show a statistical difference of adverse affects between groups.

## Limitations

Our study was powered to show a significant difference between groups; however, the design was retrospective in nature. As such, it has the disadvantage of incomplete documentation in the patient chart. Different databases were used in our institution over the study period, which could have limited consistency in documentation of patient data. This was a study in silo and not a head-to-head comparison of the two agents, and as such, baseline demographics between the two groups were not similar. In addition, we had no control over prescribing patterns according to the patient's location, medical history, and indication for laxative use. Therefore, this study encompasses real life use of these laxatives.

## Conclusion

This is the first study that supports the efficacy of oral docusate in the treatment of hospitalized pediatric patients with acute constipation. It is also the first study that demonstrates no difference in efficacy, adverse effect, or acceptance between docusate and PEG-3350.

Studies in adults have called into question the efficacy of docusate for the treatment of constipation, leading institutions to remove it from their institutional formulary. Our findings do not match those in the adult population. We believe docusate has a niche in pediatric patients, especially those who require fluid restrictions.

There seems to be a trend in selecting PEG-3350 over other laxatives, perhaps due to published literature on its efficacy. Docusate was introduced prior to FDA requirements for approval, as such there are very limited published data on its efficacy. Our study supports the utility of docusate in the management of hospitalized pediatric patients with acute constipation. Our finding underscore the need for further research in this area.

## Data Availability

The raw data supporting the conclusions of this article will be made available by the authors, without undue reservation.
